# Measurement of Microbial DNA Polymerase Activity Enables Detection and Growth Monitoring of Microbes from Clinical Blood Cultures

**DOI:** 10.1371/journal.pone.0078488

**Published:** 2013-10-14

**Authors:** Daniel R. Zweitzig, Nichol M. Riccardello, John Morrison, Jason Rubino, Jennifer Axelband, Rebecca Jeanmonod, Bruce I. Sodowich, Mark J. Kopnitsky, S. Mark O’Hara

**Affiliations:** 1 Research and Development, ZEUS Scientific, Raritan, New Jersey, United States of America; 2 St. Luke’s University Health Network, Bethlehem, Pennsylvania, United States of America; Charité-University Medicine Berlin, Germany

## Abstract

Surveillance of bloodstream infections (BSI) is a high priority within the hospital setting. Broth-based blood cultures are the current gold standard for detecting BSI, however they can require lengthy incubation periods prior to detection of positive samples. We set out to demonstrate the feasibility of using enzymatic template generation and amplification (ETGA)-mediated measurement of DNA polymerase activity to detect microbes from clinical blood cultures. In addition to routine-collected hospital blood cultures, one parallel aerobic blood culture was collected and immediately refrigerated until being transported for ETGA analysis. After refrigeration holding and transport, parallel-collected cultures were placed into a BACTEC incubator and ETGA time-course analysis was performed. Of the 308 clinical blood cultures received, 22 were BACTEC positive, and thus were initially selected for ETGA time course analysis. The ETGA assay detected microbial growth in all 22 parallel-positive blood cultures in less time than a BACTEC incubator and also yielded genomic DNA for qPCR-based organism identification. In summary, feasibility of detecting microbes from clinical blood culture samples using the ETGA blood culture assay was demonstrated. Additional studies are being considered towards development of clinically beneficial versions of this methodology.

## Introduction

Bloodstream infections (BSI) can cause sepsis, which is associated with significant patient morbidity and mortality [1,2]. The health care costs related to sepsis are also high. In fact, a recent study estimated that the annual economic burden associated with BSI care in the United States is $17 billion [2]. It should therefore not be surprising that surveillance and detection of BSI is of high priority within the hospital setting [3]. 

Broth-based blood culture is the current gold standard test for detecting BSI and is a prerequisite to additional downstream microbiology testing aimed at guiding appropriate therapy [4-8]. This approach is problematic, as sepsis can rapidly progress over the course of hours, while blood culture results can take days to confirm the presence of a hematopathogen [3,8,9]. Since delays in administration of proper antimicrobials have been shown to increase morbidity and mortality rates [10], patients are often prescribed empiric broad spectrum antimicrobial therapy with the hope of narrowing the antibiotic coverage once the pathogen is identified to the genus or species level. Unfortunately, this practice contributes to suboptimal therapy and the increasing problem of multidrug-resistant organisms through selection pressure and may also contribute to superinfections [11,12].

To address these issues, numerous rapid nucleic acid tests (NAT) have been developed with the goal of reducing the time to confirmation of BSI as well as pathogen identification (ID) and antimicrobial susceptibility determination [13-15]. Despite showing promise, adoption of NAT within the clinical microbiology laboratory has been met with resistance for numerous reasons including high costs and the inability of NAT to distinguish live microbes from circulating nucleic acid [16]. Also, studies have revealed that the broad range detection of microbes via NAT, such as 16S-targeted PCR, may not represent bona fide universal detection assays due to sequence variability across species [17-19].

We recently developed a novel methodology that enables rapid, sensitive, and universal detection of viable microbes via enzymatic template generation and amplification (ETGA)-mediated measurement of endogenous DNA polymerase activity [20]. We subsequently used a differential cell lysis procedure in combination with ETGA (referred to hereafter as the ETGA Blood Culture Assay) to enable detection of microbes 3 times faster than a continuous monitoring blood culture incubator during simulated BSI experiments [21]. Herein, we set out to verify that the ETGA Blood Culture Assay could also detect the presence and growth of microorganisms in clinical blood culture samples. 

## Materials and Methods

### Hospital Setting and Patient Enrollment

Saint Luke’s Hospital and Health Network Internal Review Board (IRB), Bethlehem PA, approved this study protocol, identification number, SLHN 2011-04. Patients were screened for enrollment at a Level 1 community trauma center with a 37-bed Intensive Care Unit (ICU), a 45-bed Emergency Department (ED), and an annual census of 75,000. Adult patients (age 18 or older) in the ED and ICU were eligible for inclusion if they presented with signs and symptoms suggestive of serious bacterial infection and if their clinical evaluation included blood cultures at the discretion of the enrolling physician. Upon determination of eligibility, subjects or their health care proxies were approached to volunteer for participation; written informed consent was obtained prior to enrollment. 

### Clinical Blood Culture Sample Collection and Subsequent Handling

Upon voluntary written consent enrollment, patients underwent a routine blood culture collection procedure as per hospital protocol. Blood culture protocol typically consists of two sets of blood culture bottles (2 aerobic BD-cat# 442192 and 2 anaerobic BD-cat# 442191) collected using aseptic technique. Blood was collected simultaneously into a third aerobic blood culture bottle which was designated only for parallel BACTEC and ETGA blood culture assay analysis (no analyses from this specimen were used in clinical decision making). Hereafter, the third parallel-collected aerobic culture bottle will be referred to as the ETGA-bottle. All 4 hospital culture bottles were incubated in BD BACTEC^TM^ automated blood culture system (BD Company, Franklin Lakes, NJ) in the hospital lab. A hospital blood culture was defined as positive using criteria outlined by the Centers for Disease Control [22], and the causative microorganism was subsequently identified according to standard clinical microbiology procedures. The ETGA-bottle was routinely drawn last in the draw sequence, blind coded, stored in a refrigerator, and transported twice-weekly 50 miles to our laboratory for independent BACTEC and ETGA analysis. 

### BACTEC and ETGA Time Course Analysis of Clinical Blood Cultures

At the initiation of this study, ideal resources were not available for around-the-clock time course ETGA monitoring of the ETGA-bottle within the hospital laboratory. Therefore, the ETGA-bottle was refrigerated prior to being transported to our laboratory for ETGA analysis. Although refrigeration is not recommended by CLISI [23], recent studies have shown that microbial growth is unaffected after limited refrigeration of blood culture samples [24]. After being transported from the hospital, the ETGA-bottle was placed into a BACTEC 9050 incubator. One milliliter aliquots were aseptically removed in a time course manner using a 3 mL syringe (21ga. Needle) and were immediately refrigerated to enable synchronization of the ETGA sample preparation procedure. For BACTEC-positive ETGA-bottles, blood culture time course aliquots were promptly removed from refrigeration holding and 0.75 mL was subjected to the ETGA blood culture assay procedure (described below). One hundred micro-liters from each of the remaining blood culture aliquots (non-processed) were also immediately plated onto blood agar for parallel CFU monitoring. After completion of the ETGA blood culture assay procedure, sample lysates were frozen at -20°C to enable subsequent nucleic acid analysis. For BACTEC-positive ETGA-bottles, microbe identification (derived from the corresponding hospital blood cultures) were retrospectively obtained from the hospitals’ microbiology laboratory.

### The ETGA Blood Culture Assay Procedure

The ETGA Blood Culture Assay consists of a differential cell lysis procedure followed by measurement of microbe-derived DNA polymerase extension activity via ETGA. A detailed description of the core ETGA technology and subsequent development of the ETGA Blood Culture Assay have been recently described [20,21]. Briefly, 0.75 mL of blood culture is transferred to a 1.5 mL tube already containing 250 uL of 1% Triton X-100. The sample tube is then capped, inverted four times to mix, and incubated for 5 minutes at room temperature. After spinning at 8000 x g for 3 minutes, the supernatant is poured off into a waste container and the tube is then inverted onto a plastic-backed lab wipe to drain any residual liquid. Using an extended pipette tip, 1 ml of 5 mM NaOH is added and subsequently pipetted up and down ten times to resuspend blood debris. The sample tube is then capped, incubated for 5 minutes at room temperature, and spun at 8000 x g for 3 minutes. The supernatant is poured off into a waste container and the tube is then inverted onto a plastic-backed lab wipe to drain any residual liquid. Next, 0.6 mL of a Tris-based wash buffer is added, pipetted up and down 5 times to resuspend the sample, and simultaneously transferred to pre-labeled bead mill lysis tube. All beadmill lysis tubes are then spun at 8000 x g for 3 minutes. After spinning, supernatants are carefully removed using a 1 mL pipette and DNA polymerase extension activity is assayed as described previously [20,21].

### Gene Specific qPCR Assays

The primers and probes for the *S. aureus* and *E. coli*-specific assays have been previously reported [20]. The primer and probe sequences for the *S. agalactiae* and *S. epidermidis* assays are as follows: *S. agalactiae* - Forward primer 5’ –TCGCATTTTAGATCCATTTGC- 3’, Reverse primer 5’ –GCTCTATCAGTTGGTTTTAAATCAGG- 3’, Probe 5’ –CAATTAAAGCTCAAG TTAACGATGTAAAGGCA 3’. *S. epidermidis* - Forward primer 5’ –CAACTCGATGCA AATCAGCAA- 3’, Reverse primer 5’ - GAACCGCATAGCTCCCTGC- 3’, Probe 5’ - TACTACGCTGGTGGAACTTCAAA TCGTTATCG- 3’. The qPCR reactions were prepared as follows: 300nM forward and reverse primers, 100nM hydrolysis probe, 15µL Light Cycler 480 Probes Master (Roche Applied Sciences cat# 04902343001), nuclease free water (Life Technologies, cat# AM9932) to a volume of 26µL, and 4µL of sample. The samples were analyzed on a Cepheid Smart Cycler II (Sunnyvale, CA) using the following protocol: 95°C for 5 minutes, and then 45 cycles of 95° for 15 seconds followed by 60°C for 1 minute. Fluorescence was measured during the 60°C stage of each cycle.

## Results and Discussion

### BACTEC Results and Corresponding Microbial ID

**Figure 1:**
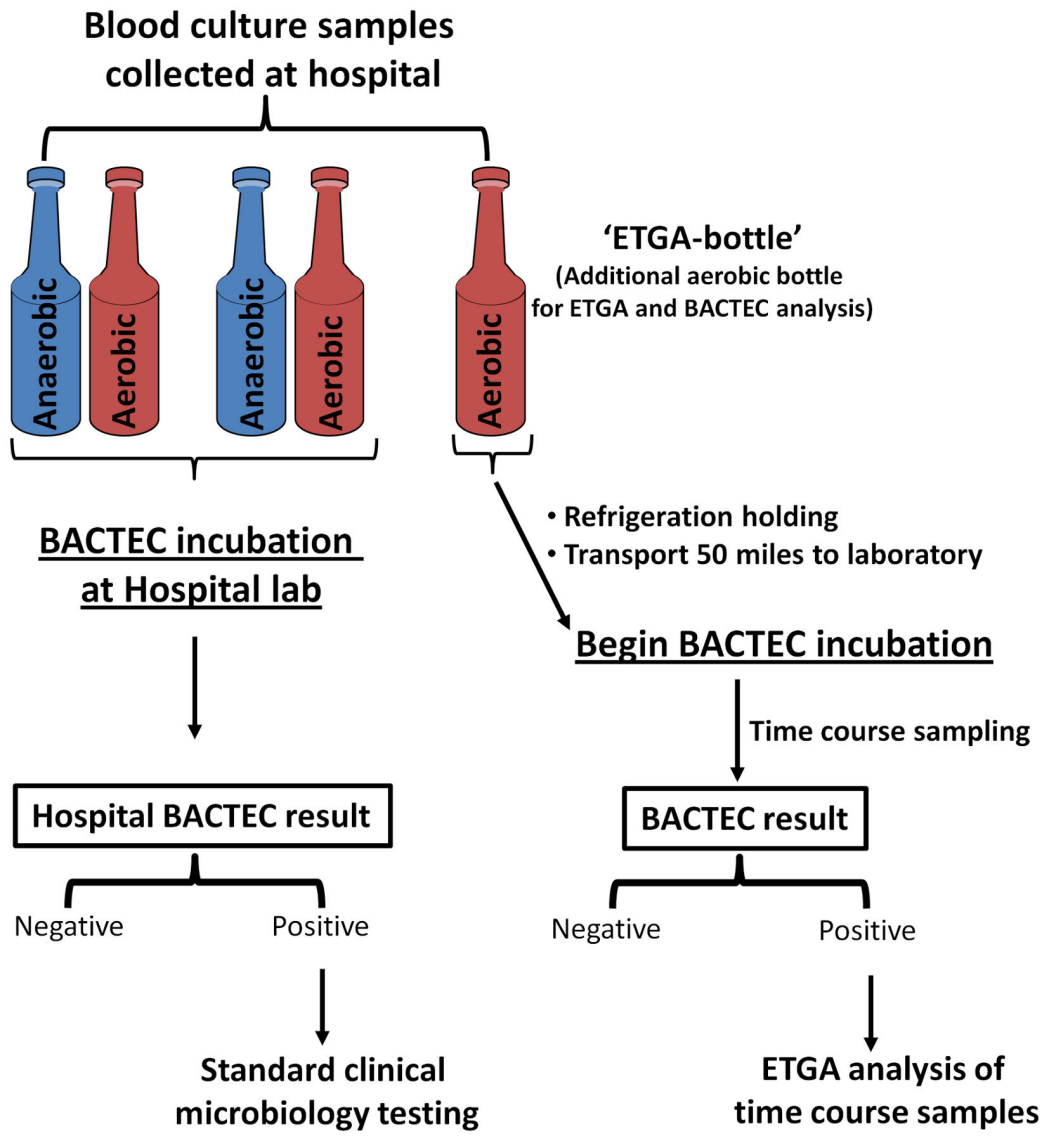
A schematic overview of the study design is presented.

In this report, we set out to assess the feasibility of detecting microbes from clinical blood culture samples via selective measurement of their DNA polymerase extension activity using ETGA. A schematic diagram of the proposed experimental approach is presented in Figure 1. Initially, 308 ETGA-bottles were collected and subjected to BACTEC analysis. Of these, 22 yielded positive BACTEC results (Table 1). The microbial ID for each of these 22 samples was obtained retrospectively from the parallel-collected blood culture samples that were analyzed by the hospital laboratory, and are also presented in Table 1. The most prevalent organisms found to be associated with the BACTEC-positive samples were *E. coli* and *Streptococcus sp.*, followed by *Staphylococcus* *sp*. Consistent with previous observations, these species are common among BSI-causing microorganisms [14]. 

**Table 1:** BACTEC Time-to-Detection (TTD) for ETGA-bottles and the corresponding hospital-derived microbe identification.

**Sample ID**	**Hospital Microbe ID**	**BACTEC Result (TTD hours)**
4	*E. coli*	Positive (11.67)
12	*E. coli*	Positive (10.83)
39	*E. coli*	Positive (14.83)
63	*E. coli*	Positive (10.67)
183	*E. coli*	Positive (13.17)
254	*E. coli*	Positive (10.67)
256	*E. coli*	Positive (11.00)
208	*S. aureus*	Positive (12.33)
258	*S. aureus*	Positive (10.67)
273	*S. aureus*	Positive (8.33)
190	*S. aureus*	Positive (15.17)
255	CoNS	Positive (22.67)
165	CoNS	Positive (26.83)
5	Beta h strep - Group A	Positive (11.00)
210	Beta h strep - Group C	Positive (11.83)
217	Beta h strep - Group B	Positive (13.33)
289	Beta h strep - Group G	Positive (19.83)
86	Proteus	Positive (13.33)
114	Abiotrophia/granulicatella	Positive (30.67)
229	Alpha haemolytic strep	Positive (22.67)
95	*S. pneumoniae*	Positive (14.33)
301	*S. pneumoniae*	Positive (14.83)

BACTEC TTD values are presented for the ETGA-bottles. Microbial ID’s provided by parallel analyses of the corresponding cultures at the Hospital’s clinical microbiology laboratory are also presented.

### Growth Monitoring of Clinical Blood Cultures via the ETGA Blood Culture Assay

**Figure 2:**
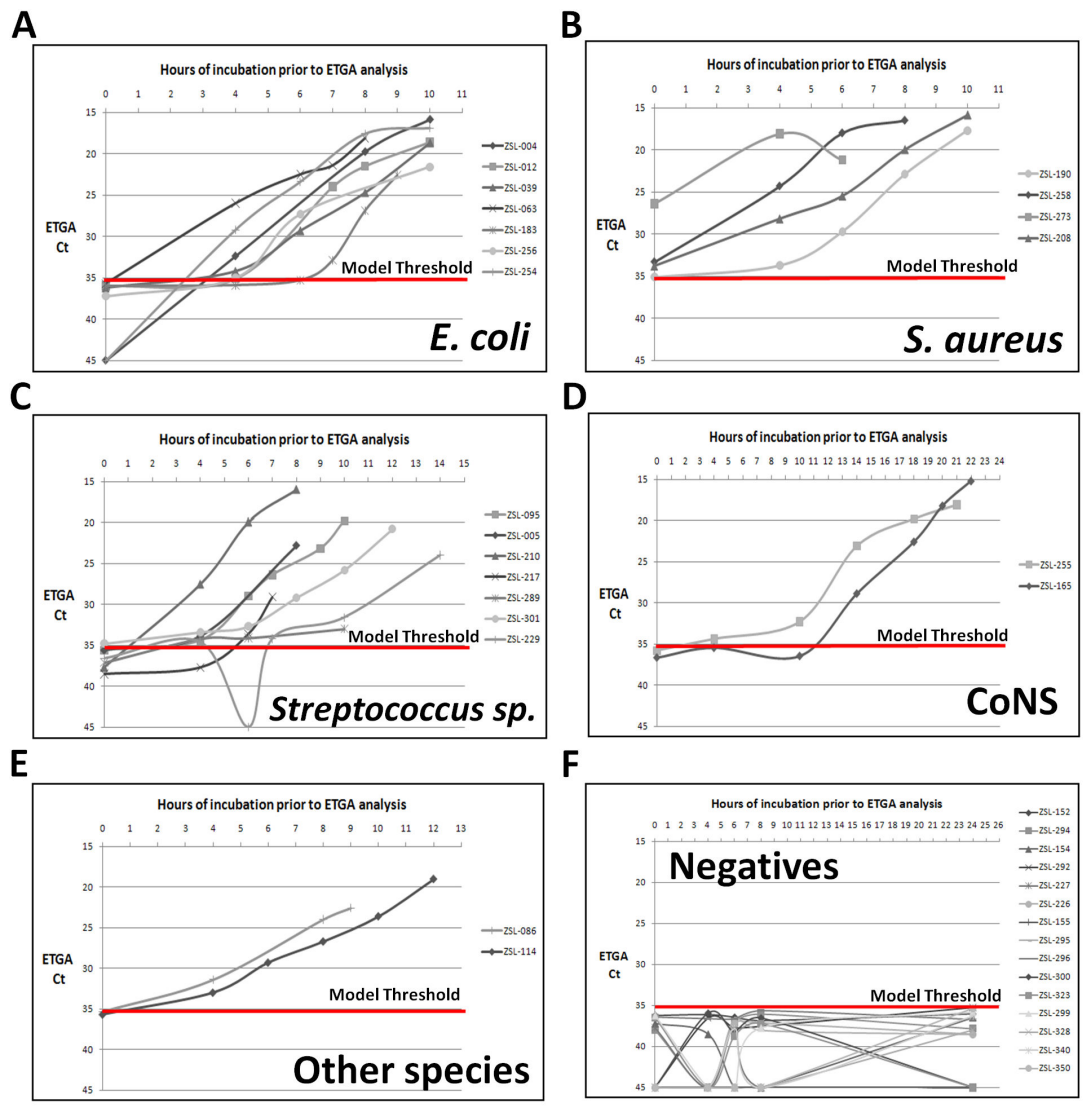
ETGA time course monitoring of clinical blood cultures. (A-E) BACTEC-positive ETGA-bottles that underwent time course monitoring were separated into 5 groups and linear plots were generated using their respective ETGA Blood Culture Assay Ct values versus the time each culture bottle spent within the BACTEC instrument. A horizontal red line, representing a previously determined ETGA blood culture assay positivity threshold, is also included in each plot as a visual reference. The sample ID numbers are also included to the right of the curves in each graph and can be used to locate the corresponding microbial ID and BACTED TTD values using Table 1. (F) Linear plots are also presented from ETGA time course analysis of 15 BACTEC-negative ETGA-bottles.

As mentioned, the primary goal of this study was to assess the feasibility of using the ETGA blood culture assay to detect the presence and growth of microbes within clinical blood cultures. Towards this goal, ETGA blood culture assay time course analysis was performed for each of the 22 ETGA-bottles that were called positive by the BACTEC instrument. The ETGA assay time course analysis data for these samples were assembled into 5 separate groups and are presented in Figures 2A-E. A horizontal red line was incorporated into each graph, representing a positivity threshold cycle threshold (Ct) value of 35.24 that was previously established from ETGA Blood Culture Assay analysis of blood cultures obtained from healthy donors [21]. Despite being determined previously, the 35.24 threshold Ct marker is included as a visual reference within the time course graphs to help differentiate microbial growth from typical blood culture sample background noise. As shown in Figures 2A-E, the ETGA blood culture assay sensitively detected the presence and growth of microbes within all 22 of the ETGA-bottles. The ETGA Ct values and parallel CFU plating data for these samples also correlate well with one another (Tables S2-S6). Of note, the growth curve derived from sample ZSL-229 dipped below the positivity threshold at the 6 hour time point (Figure 2C). This atypical growth curve behavior correlates well with the CFU fluctuation and/or mixed microbial population observed from parallel plating analysis of sample aliquots taken from the corresponding ETGA-bottle (Figure S1). 

Taken together, these preliminary time course studies suggest that the ETGA blood culture assay has potential to reduce the Time-to-Detection (TTD) of microbes from clinical samples, when compared to standard BACTEC analysis (Table S1). In fact, ETGA detected 100% (4 of 4) of the samples containing *S. aureus* at time zero (Figure 2B). Also, considering the range of bacterial species present (Table 1), these data support the potential universality of detecting microbes from clinical samples via the highly conserved biochemical activity of DNA polymerase extension. Furthermore, when considering these clinical samples and previously published data obtained during assay development studies [20,21], ETGA has detected without fail more than 30 different microbial species. 

In addition to the 22 positive samples, time course aliquots from 15 BACTEC-negative ETGA-bottles were subjected to ETGA blood culture assay time course analysis. As shown in Figure 2F, all of the ETGA blood culture assay signals derived from the 15 negative clinical samples remained below the positivity Ct threshold, and are thus consistent with the previously determined healthy donor threshold [21]. Therefore, these results demonstrate that the growth curves presented in Figures 2A-E are specific for microbial growth and not reflective of artifactual changes in assay background signal due to the time that these negative blood cultures spent in refrigeration and/or in the BACTEC incubator. For more accurate clinical diagnostic positivity/negativity and ETGA TTD determinations, a rigorous threshold value will need to be determined statistically using ETGA assay signals derived from a larger number of negative clinical blood culture samples that had not been subjected to atypical handling such as refrigeration. 

### Nucleic Acid-Based Microbial Identification from ETGA Lysates

In addition to DNA polymerase activity, we previously demonstrated that ETGA lysates from simulated BSI also contain microbe-derived nucleic acid, and thus are readily available for subsequent microbial identification via rapid NAT such as qPCR [21]. To determine the feasibility of parallel NAT analysis of clinical blood cultures, we retrospectively interrogated the ETGA lysates (generated from 13 of the 22 BACTEC-positive ETGA-bottles) for the genomic DNA of *S. aureus*, *E. coli*, *S.* epidermidis, and *S. agalactiae*, based upon availability of the corresponding in-house gene specific qPCR (gsPCR) assays. The gsPCR ID results presented in Table 2 agree with the Hospital microbiology laboratory’s independent ID, and demonstrate that the ETGA blood culture assay procedure produced lysates from clinical samples that are suitable for rapid parallel nucleic acid-based species identification. 

**Table 2:** Bacterial identification from ETGA assay sample lysates using gene specific PCR.

**Sample ID**	**Hospital Microbe ID**	***S. aureus* Assay Result**	***E. coli* Assay Result**	***S. epidermidis* Assay Result**	***S. agalactiae* Assay Result**
4	*E. coli*	Negative	Positive	Negative	Negative
12	*E. coli*	Negative	Positive	Negative	Negative
39	*E. coli*	Negative	Positive	Negative	Negative
63	*E. coli*	Negative	Positive	Negative	Negative
183	*E. coli*	Negative	Positive	Negative	Negative
256	*E. coli*	Negative	Positive	Negative	Negative
254	*E. coli*	Negative	Positive	Negative	Negative
208	*S. aureus*	Positive	Negative	Negative	Negative
190	*S. aureus*	Positive	Negative	Negative	Negative
258	*S. aureus*	Positive	Negative	Negative	Negative
273	*S. aureus*	Positive	Negative	Negative	Negative
217	Streptococcus-group B	Negative	Negative	Negative	Positive
165	*S. epidermidis*	Negative	Negative	Positive	Negative

Thirteen of the ETGA Blood Culture Assay Sample lysates were analyzed by 4 different qPCR assays specific for genomic DNA targets of *S. aureus*, *E. coli*, *S.* epidermidis, and *S. agalactiae*.

## Summary

Herein, we demonstrated the feasibility of using the ETGA blood culture assay to rapidly detect the presence and growth of microbes within clinical blood cultures. Together, we feel that these results merit additional studies aimed at performing time course analysis of clinical blood cultures within the hospital laboratory setting, towards a more realistic assessment of the ETGA blood culture assay’s capability to be used as a rapid BSI screening tool. However, in its current manual form, it would be difficult to incorporate the ETGA blood culture assay into the busy workflow of the modern clinical microbiology laboratory. To this end, experiments are underway to further simplify the ETGA blood culture assay and make it more amenable to automation. If achieved, automation could potentially reduce the TTD of BSI. Also, an ETGA lysate-coupled NAT approach could enable much faster organism identification times and initiation of data-guided antimicrobial susceptibility testing faster than traditional methods. The importance of this proposed approach is supported by the mounting evidence that rapid identification of BSI leads to lower morbidity and mortality, translating into reduced length of hospital stay and associated cost savings [25]. We are also interested in evaluating procedural modifications that would enable ETGA-mediated detection of microbes directly from whole blood and blood products.

## Supporting Information

Figure S1 **The plating images and ETGA curves are presented for sample ZSL-229 in an effort to highlight the potential source of the atypical growth curve behavior presented in Figure 2C.**
(TIF)

Table S1 **Time-to-Detection comparisons were compared for the ETGA blood culture assay versus a BACTEC instrument.**
(TIF)

Table S2 **The raw ETGA data and parallel CFU monitoring of the time course results corresponding to Figure 2A.**
(TIF)

Table S3 **The raw ETGA data and parallel CFU monitoring of the time course results corresponding to Figure 2B.**
(TIF)

Table S4 **The raw ETGA data and parallel CFU monitoring of the time course results corresponding to Figure 2C.**
(TIF)

Table S5 **The raw ETGA data and parallel CFU monitoring of the time course results corresponding to Figure 2D.**
(TIF)

Table S6 **The raw ETGA data and parallel CFU monitoring of the time course results corresponding to Figure 2E.**
(TIF)
