# Supplementary figures and images for: Measurement of Microbial DNA Polymerase Activity Enables Detection and Growth Monitoring of Microbes from Clinical Blood Cultures
Source: PLoS One. 2013 Oct 14;8(10):e78488. doi: 10.1371/journal.pone.0078488 (PMC3796490; doi:10.1371/journal.pone.0078488)

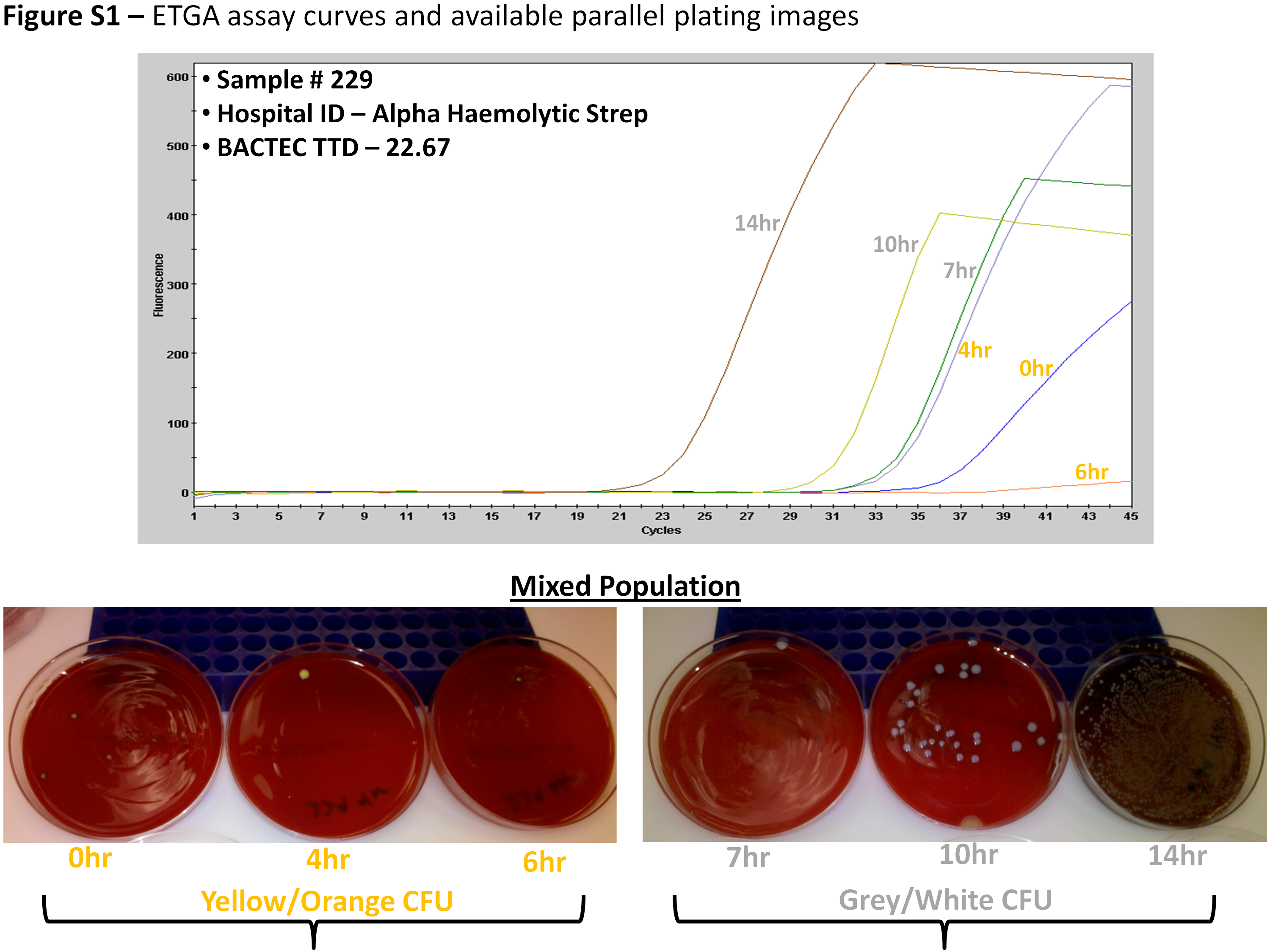

Supplement: Figure S1 — The plating images and ETGA curves are presented for sample ZSL-229 in an effort to highlight the potential source of the atypical growth curve behavior presented in Figure 2C. (TIF) [file pone.0078488.s001.tif]
